# Supplementary material for: Mental Health and Quality of Life of Women One Year after Maternal Near-Miss in Low and Middle-Income Countries: The Case of Zanzibar, Tanzania
Source: Int J Environ Res Public Health. 2020 Dec 3;17(23):9034. doi: 10.3390/ijerph17239034 (PMC7730062; doi:10.3390/ijerph17239034)
Supplement: Supplementary file 1 [file ijerph-17-09034-s001.pdf]

Supplementary Tables

Table S1: Adjusted maternal near-miss criteria for Mnazi Mmoja Hospital in Zanzibar.

Type of organ dysfunction	Markers (clinical signs, laboratory markers and management actions)
**Cardiovascular**	Shock[1], use of continuous vasoactive drugs, cardiac arrest, cardio-pulmonary resuscitation, severe hypoperfusion (lactate >5 mmol/L or >45mg/dL)2 or severe acidosis (pH <7.1)[2]
**Respiratory**	Acute cyanosis, gasping[3], severe tachypnea (respiratory rate >40bpm), severe bradypnea (respiratory rate <6bpm), severe hypoxemia (PAO2/FiO2 <200mmHg2 or O2 saturation <90% for ≥60min) or intubation and ventilation not related to anaesthesia
**Renal**	Oliguria[4] non-responsive to fluids or diuretics, dialysis for acute renal failure[5] or severe acute azotaemia (creatinine ≥300umol/ml or ≥3.5mg/dL)
**Coagulation / haematological**	Clotting failure[6], use of continuous vasoactive drugs[7], massive transfusion of blood or red cells (≥5 units)[8] or severe acute thrombocytopenia (<50,000 platelets/ml)
**Hepatic**	Jaundice in the presence of pre-eclampsia[9], severe acute hyperbilirubinemia (bilirubin >100umol/L or >6.0mg/dL)
**Neurological**	Prolonged unconsciousness (lasting >12 hours)/coma[10], stroke[11], status epilepticus[12], uncontrollable fits/total paralysis
**Uterine**	Hysterectomy following haemorrhage or infection

Unless otherwise stated, the criteria are reproduced from Herklots et al. 2019 [a]:

1 Shock is a persistent severe hypotension, defined as a systolic blood pressure <90 mmHg for ≥60 minutes with a pulse rate at least 120 despite aggressive fluid replacement (>2l)
2 Laboratory test or management intervention that is not available at MMH
3 Gasping is a terminal respiratory pattern and the breath is convulsively and audibly caught
4 Oliguria is defined as a urinary output <30 ml/hr for 4 hours or <400 ml/24 hr
5 Dialysis services have been available in MMH since 26 May 2017
6 Clotting failure can be assessed by the bedside clotting test or absence of clotting from the intravenous site after 7–10 minutes
7 For instance, continuous use of any dose of dopamine, epinephrine or norepinephrine
8 In MMH extended to include all types of blood products and cases in which 5 or more units were requested but not given due to shortage
9 Pre-eclampsia is defined as the presence of hypertension associated with proteinuria. Hypertension is defined as a blood pressure of at least 140 mmHg (systolic) or at least 90 mmHg (diastolic) on at least two occasions and at least 4–6 h apart after the 20th week of gestation in women known to be normotensive beforehand. In MMH, proteinuria is defined as ≥2+ protein on dipstick.
10 Loss of consciousness is a profound alteration of mental state that involves complete or near-complete lack of responsiveness to external stimuli. It is defined as a Coma Glasgow Scale <10 (moderate or severe coma).
11 Stroke is a neurological deficit of cerebrovascular cause that persists beyond 24 hours or is interrupted by death within 24 hours
12 Condition in which the brain is in a state of continuous seizure

[a] Herklots T, van Acht L, Khamis RS, Meguid T, Franx A, Jacod B. Validity of WHO's near-miss approach in a high maternal mortality setting. PLOS ONE. 2019;14(5):e0217135

Table S2. Intercurrent illness of MNM and control women overtime.

Intercurrent illness	3-month		6-month		12-month		Total	
	MNM	Control	MNM	Control	MNM	Control	MNM	Control
**Yes**	41 (0.304)	23 (0.168)	24 (0.229)	21 (0.196)	46 (0.368)	18 (0.164)	62 (19.6)	111 (27.5)
**No**	94 (0.696)	114 (0.832)	86 (0.771)	86 (0.804)	79 (0.632)	92 (0.836)	254 (80.4)	292 (72.2)
**Total**	135(0.504)	137 (0.496)	105 (0.505)	107 (0.495)	125 (0.468)	110 (0.532)	316 (43.9)	403 (56.1)

Table S3. Baseline demographic and clinical characteristics of participants who take part in the follow-up and those who lost to follow-up.

		Lost to follow-up			
		Yes	No	Total	p-value
**Age**	Mean (SD)	28.4 (6.6)	28.9 (5.6)	28.8 (5.9)	0.234
**Marital status**	Married	80 (89.9)	305 (92.4)	385 (91.9)	0.437
	Others	9 (10.1)	25 (7.6)	34 (8.1)	
**Address**	Mixed	12 (46.2)	59 (39.9)	71 (40.8)	0.800
	Rural	8 (30.8)	47 (31.8)	55 (31.6)	
	Urban	6 (23.1)	42 (28.4)	48 (27.6)	
**Education**	Primary or less	31 (36.9)	116 (35.7)	147 (35.9)	0.836
	Secondary or more	53 (63.1)	209 (64.3)	262 (64.1)	
**Employment**	Employed	8 (9.5)	56 (17.0)	64 (15.5)	0.209
	Housewife	43 (51.2)	176 (53.5)	219 (53.0)	
	Self-employed	28 (33.3)	82 (24.9)	110 (26.6)	
	Others	5 (6.0)	15 (4.6)	20 (4.8)	
**Perceived wealth**	Average	65 (77.4)	263 (83.2)	328 (82.0)	0.215
	Below average	19 (22.6)	53 (16.8)	72 (18.0)	
**Gestational age**	First trimester	7 (8.6)	29 (12.1)	36 (11.2)	0.230
	Second trimester	7 (8.6)	10 (4.2)	17 (5.3)	
	Third trimester	67 (82.7)	201 (83.8)	268 (83.5)	
**Mode of delivery**	Vaginal delivery	36 (27.5)	98 (30.0)	134 (29.3)	0.135
	Cesarean section	83 (63.4)	179 (54.7)	262 (57.2)	
	Early pregnancy loss	12 (9.2)	50 (15.3)	62 (13.5)	
**Parity**	1	64 (48.5)	149 (44.0)	213 (45.2)	0.427
	2-4	44 (33.3)	135 (39.8)	179 (38.0)	
	>4	24 (18.2)	55 (16.2)	79 (16.8)	
**History of cesarean section**	One or more	15 (26.3)	43 (24.0)	58 (24.6)	0.726
	No	42 (73.7)	136 (76.0)	178 (75.4)	

Table S4: Result for an individual item of questionnaires (PHQ-9, HTQ-16, PSS, and WHOQOL-BREEF).

		3 Months		6 months		12 months	
	Total	Control	MNM	Control	MNM	Control	MNM
**PHQ-9 Items Mean (SD)**	**(N = 715)**	**(N = 141)**	**(N = 147)**	**(N = 106)**	**(N = 111)**	**(N = 88)**	**(N = 122)**
Little interest or pleasure in doing things	0.09 (0.33)	0.09 (0.29)	0.14 (0.41)	0.01 (0.10)	0.10 (0.33)	0.07 (0.37)	0.09 (0.36)
Feeling down, depressed, or hopeless	0.15 (0.46)	0.21 (0.53)	0.28 (0.62)	0.12 (0.41)	0.16 (0.48)	0.03 (0.18)	0.02 (0.20)
Trouble falling or staying asleep, or sleeping too much	0.14 (0.47)	0.15 (0.49)	0.12 (0.39)	0.09 (0.38)	0.17 (0.55)	0.08 (0.38)	0.20 (0.58)
Feeling tired or having little energy	0.65 (0.72)	0.60 (0.65)	0.63 (0.73)	0.59 (0.61)	0.64 (0.66)	0.63 (0.83)	0.80 (0.83)
Poor appetite or overeating	0.36 (0.74)	0.30 (0.64)	0.29 (0.69)	0.38 (0.76)	0.42 (0.76)	0.38 (0.82)	0.42 (0.80)
Feeling bad about yourself - or that you are a failure or have let yourself or your family down	0.15 (0.45)	0.21 (0.49)	0.21 (0.54)	0.07 (0.37)	0.12 (0.35)	0.08 (0.31)	0.14 (0.52)
Trouble concentrating on things, such as reading the newspaper or watching television	0.06 (0.31)	0.06 (0.31)	0.14 (0.48)	0.04 (0.24)	0.03 (0.16)	0.02 (0.15)	0.06 (0.30)
Moving or speaking so slowly that other people could have noticed Or, the opposite	0.03 (0.23)	0.04 (0.22)	0.07 (0.42)	0.00 (0.00)	0.02 (0.14)	0.00 (0.01)	0.02 (0.18)
Thoughts that you would be better off dead or of hurting yourself in some way	0.06 (0.34)	0.09 (0.37)	0.14 (0.54)	0.00 (0.02)	0.05 (0.25)	0.03 (0.32)	0.03 (0.22)
**HTQ-16 Items Mean (SD)**	**(N = 719)**	**(N = 140)**	**(N = 145)**	**(N = 107)**	**(N = 112)**	**(N = 93)**	**(N = 122)**
Recurrent thoughts or memories of the most hurtful or terrifying events	1.12 (0.44)	1.12 (0.42)	1.19 (0.55)	1.08 (0.30)	1.12 (0.42)	1.08 (0.40)	1.11 (0.44)
Feeling as though the event is happening again	1.07 (0.31)	1.14 (0.36)	1.14 (0.49)	1.00 (0.00)	1.06 (0.28)	1.03 (0.18)	1.02 (0.13)
Recurrent nightmares	1.06 (0.32)	1.07 (0.33)	1.10 (0.38)	1.03 (0.17)	1.05 (0.26)	1.04 (0.33)	1.07 (0.36)
Feeling detached or withdrawn from people	1.06 (0.30)	1.07 (0.31)	1.07 (0.35)	1.04 (0.19)	1.03 (0.16)	1.03 (0.31)	1.09 (0.36)
Unable to feel emotions	1.05 (0.24)	1.07 (0.28)	1.04 (0.23)	1.02 (0.14)	1.05 (0.23)	1.01 (0.10)	1.08 (0.33)
Feeling jumpy, easily startled	1.22 (0.49)	1.26 (0.50)	1.27 (0.58)	1.12 (0.36)	1.21 (0.45)	1.16 (0.50)	1.25 (0.50)
Difficulty concentrating	1.07 (0.33)	1.11 (0.42)	1.09 (0.35)	1.03 (0.22)	1.06 (0.23)	1.01 (0.10)	1.12 (0.43)
Trouble sleeping	1.09 (0.33)	1.11 (0.40)	1.12 (0.36)	1.05 (0.21)	1.09 (0.34)	1.06 (0.29)	1.09 (0.32)
Feeling on guard	1.04 (0.23)	1.06 (0.34)	1.08 (0.30)	1.01 (0.10)	1.03 (0.16)	1.03 (0.18)	1.02 (0.13)
Feeling irritable or having outburst of anger	1.09 (0.37)	1.13 (0.36)	1.12 (0.47)	1.05 (0.32)	1.06 (0.28)	1.02 (0.15)	1.12 (0.46)
Avoiding activities that remind you of the hurtful or terrifying events	1.08 (0.32)	1.08 (0.36)	1.08 (0.29)	1.03 (0.17)	1.13 (0.43)	1.06 (0.25)	1.07 (0.32)
Inability to remember parts of the hurtful or terrifying events	1.06 (0.28)	1.06 (0.23)	1.10 (0.39)	1.03 (0.17)	1.05 (0.35)	1.01 (0.10)	1.07 (0.28)
Less interest in daily activities	1.08 (0.30)	1.13 (0.36)	1.12 (0.36)	1.02 (0.14)	1.07 (0.29)	1.02 (0.15)	1.07 (0.33)
Feeling as if you don't have a future	1.04 (0.22)	1.04 (0.24)	1.03 (0.22)	1.01 (0.10)	1.01 (0.09)	1.02 (0.15)	1.08 (0.38)

Avoiding thoughts or feelings associated with the hurtful or terrifying events	1.05 (0.27)	1.07 (0.32)	1.06 (0.27)	1.04 (0.23)	1.08 (0.38)	1.01 (0.10)	1.02 (0.13)
Sudden emotional or physical reaction when reminded of the traumatic events	1.08 (0.28)	1.13 (0.36)	1.06 (0.26)	1.06 (0.23)	1.08 (0.27)	1.06 (0.25)	1.08 (0.28)
**Perceived Social Support Scale Items Mean (SD)**	**(N = 730)**	**(N = 141)**	**(N = 143)**	**(N = 103)**	**(N = 111)**	**(N = 111)**	**(N = 121)**
I get visits from friends and relatives	3.80 (0.63)	3.74 (0.65)	3.72 (0.74)	3.67 (0.80)	3.73 (0.74)	4.00 (0.00)	3.93 (0.40)
I get useful advice about important things in life	3.80 (0.62)	3.63 (0.80)	3.69 (0.75)	3.79 (0.60)	3.77 (0.74)	3.99 (0.09)	4.00 (0.00)
I am able to talk to someone about problems at work or with my housework	3.67 (0.83)	3.48 (1.00)	3.50 (1.02)	3.58 (0.87)	3.58 (0.93)	3.97 (0.16)	3.97 (0.18)
I am able to talk to someone I trust about my personal and family problems	3.69 (0.76)	3.50 (0.92)	3.52 (0.93)	3.57 (0.82)	3.67 (0.82)	3.96 (0.30)	3.99 (0.09)
I get help with money in an emergency	3.63 (0.91)	3.36 (1.12)	3.46 (1.08)	3.43 (1.04)	3.59 (0.98)	4.00 (0.00)	4.00 (0.00)
I get help when I am sick	3.78 (0.65)	3.60 (0.87)	3.73 (0.68)	3.66 (0.81)	3.76 (0.65)	4.00 (0.00)	4.00 (0.00)
I have people who care what happens to me	3.87 (0.48)	3.68 (0.71)	3.84 (0.58)	3.87 (0.39)	3.86 (0.50)	3.99 (0.09)	4.00 (0.00)
I get love and affection	3.91 (0.39)	3.83 (0.55)	3.86 (0.53)	3.91 (0.32)	3.89 (0.42)	4.00 (0.00)	4.00 (0.00)
**WHOQOL-BREEF Items Mean (SD)**							
**Physical domain**	**N = 725**	**N = 140**	**N = 142**	**N = 105**	**N = 109**	**N = 107**	**N = 122**
To what extent do you feel that physical pain prevents you from doing what you need to do?	4.43 (0.97)	4.49 (0.96)	4.43 (1.02)	4.52 (0.88)	4.31 (1.02)	4.41 (1.10)	4.41 (0.85)
How much do you need any medical treatment to function in your daily life?	4.28 (0.96)	4.37 (0.90)	4.24 (0.87)	4.21 (1.06)	4.26 (0.95)	4.26 (1.13)	4.33 (0.87)
you have enough energy for everyday life?	4.31 (1.06)	4.18 (1.13)	3.76 (1.31)	4.45 (0.93)	4.26 (0.98)	4.66 (0.75)	4.70 (0.73)
How well are you able to get around?	4.67 (0.75)	4.55 (0.83)	4.57 (0.82)	4.50 (0.91)	4.65 (0.76)	4.89 (0.54)	4.88 (0.44)
How satisfied are you with your sleep?	4.65 (0.74)	4.52 (0.80)	4.56 (0.85)	4.57 (0.72)	4.65 (0.69)	4.82 (0.63)	4.80 (0.64)
How satisfied are you with your ability to perform your daily living activities?	4.64 (0.74)	4.56 (0.81)	4.46 (0.90)	4.55 (0.75)	4.61 (0.79)	4.94 (0.33)	4.78 (0.55)
How satisfied are you with your capacity for work?	4.61 (0.78)	4.54 (0.83)	4.30 (0.97)	4.68 (0.60)	4.50 (0.91)	4.85 (0.53)	4.86 (0.50)
**Psychological domain**	**N = 725**	**N = 140**	**N = 142**	**N = 105**	**N = 109**	**N = 107**	**N = 122**
How much do you enjoy life?	4.59 (0.89)	4.42 (0.95)	4.42 (1.05)	4.45 (1.06)	4.61 (0.84)	4.83 (0.61)	4.85 (0.52)
To what extent do you feel your life to be meaningful?	4.67 (0.78)	4.48 (0.93)	4.46 (1.02)	4.57 (0.82)	4.72 (0.65)	4.95 (0.35)	4.95 (0.28)
How well are you able to concentrate?	4.72 (0.71)	4.58 (0.83)	4.57 (0.93)	4.67 (0.73)	4.72 (0.64)	4.95 (0.29)	4.89 (0.44)
Are you able to accept your bodily appearance?	4.78 (0.66)	4.77 (0.68)	4.65 (0.92)	4.81 (0.57)	4.78 (0.61)	4.91 (0.45)	4.81 (0.54)
How satisfied are you with yourself?	4.63 (0.75)	4.49 (0.83)	4.40 (0.89)	4.65 (0.65)	4.54 (0.91)	4.89 (0.40)	4.87 (0.50)
How often do you have negative feelings such as a blue mood, despair, anxiety, depression?	4.66 (0.71)	4.47 (0.88)	4.56 (0.81)	4.81 (0.42)	4.61 (0.68)	4.73 (0.75)	4.84 (0.48)

Social domain	N = 725	N = 140	N = 142	N = 105	N = 109	N = 107	N = 122
How satisfied are you with your personal relationships?	4.78 (0.54)	4.70 (0.55)	4.73 (0.56)	4.72 (0.51)	4.73 (0.70)	4.92 (0.45)	4.87 (0.41)
How satisfied are you with your sex life?	3.71 (1.90)	3.35 (2.02)	2.96 (2.10)	3.65 (1.95)	3.99 (1.65)	4.32 (1.61)	4.25 (1.51)
How satisfied are you with the support you get from your friends?	4.64 (0.87)	4.56 (0.92)	4.53 (1.00)	4.42 (1.09)	4.55 (1.01)	4.92 (0.37)	4.88 (0.40)
**Environmental domain**	**N = 725**	**N = 140**	**N = 142**	**N = 105**	**N = 108**	**N = 107**	**N = 122**
How safe do you feel in your daily life?	4.75 (0.69)	4.64 (0.83)	4.60 (0.91)	4.69 (0.74)	4.77 (0.54)	4.95 (0.29)	4.88 (0.41)
How healthy is your physical environment?	4.64 (0.83)	4.49 (0.99)	4.42 (1.05)	4.68 (0.73)	4.69 (0.82)	4.92 (0.38)	4.74 (0.63)
Have you enough money to meet your needs?	3.17 (0.99)	3.24 (1.03)	3.06 (1.03)	3.35 (0.91)	2.97 (0.83)	3.32 (1.08)	3.09 (0.97)
How available to you is information that you need in your day-to-day life?	4.73 (0.73)	4.64 (0.80)	4.61 (0.96)	4.74 (0.63)	4.72 (0.69)	4.85 (0.58)	4.85 (0.46)
To what extent do you have the opportunity for leisure activities?	4.56 (0.96)	4.35 (1.18)	4.28 (1.23)	4.53 (0.83)	4.62 (0.79)	4.87 (0.65)	4.82 (0.53)
How satisfied are you with the conditions of your living place?	4.65 (0.74)	4.51 (0.80)	4.49 (0.89)	4.55 (0.82)	4.65 (0.75)	4.93 (0.32)	4.83 (0.49)
How satisfied are you with your access to health services?	4.61 (0.83)	4.45 (0.94)	4.45 (1.02)	4.50 (0.87)	4.56 (0.89)	4.90 (0.35)	4.84 (0.49)
How satisfied are you with your transport?	4.49 (0.94)	4.20 (1.09)	4.38 (1.01)	4.38 (0.99)	4.47 (1.07)	4.86 (0.44)	4.74 (0.61)
**Extra questions**							
How would you rate your quality of life?	3.05 (0.63)	3.08 (0.62)	2.98 (0.65)	3.06 (0.54)	2.95 (0.52)	3.25 (0.81)	2.98 (0.54)
How satisfied are you with your health?	3.95 (0.92)	4.06 (0.96)	3.99 (0.92)	3.96 (0.80)	3.96 (0.82)	4.02 (1.00)	3.70 (0.95)

Table S5: Output of HTQ-16 model with exponentiated estimates.

	EXP (Estimate)	EXP (95% CI)		p-value
		lower	upper	
(Intercept)	1.20	0.87	1.66	0.260
Time after discharge from hospital	1.09	1.02	1.16	0.013
MNM status (near-miss women)	1.14	0.72	1.78	0.579
MNM status * time after discharge	0.90	0.83	0.98	0.020

Table S6: Output of PHQ-9 model with un-exponentiated estimates. [a,b,c]

	Estimate	z.value	95% CI		p-value
			lower	upper	
(Intercept)	1.62	4.99	0.98	2.25	0.000
Time after discharge from hospital	0.00	-0.14	-0.04	0.03	0.888
MNM status (near-miss women)	0.04	0.32	-0.23	0.32	0.750
Pregnancy outcome (Early pregnancy loss)	-0.09	-0.55	-0.41	0.23	0.582
Pregnancy outcome (Perinatal loss)	0.26	2.04	0.01	0.51	0.041
Intercurrent illness of mother (yes)	0.35	3.25	0.14	0.56	0.001
Social support scale	-0.05	-4.69	-0.07	-0.03	0.000
MNM status * time after discharge	0.01	0.38	-0.04	0.05	0.705

[a] The "intercept" shows the initial status (three months) depressive symptom score for a control woman if all other variables are zero. The hypothesis tested here (p-value) is if it is different from zero or not. "Time after discharge from hospital" is the slope (rate of change in depressive symptom) for controls, and the hypothesis tested here (p-value) is if the slope is different from zero or not (if there is change over time or not). Whereas, the interaction between time after discharge and MNM status ("MNM status * time after discharge") indicates if the slope of MNM women differs significantly from the controls. Lastly, "MNM status (near-miss women)" shows if the initial status of depressive symptoms of MNM women differs significantly from controls.

[b] Three months are subtracted from the variable "time after discharge from hospital."

[c] The reference category for variables in the model is as follows: MNM status (control women), Pregnancy outcome (baby alive), and Intercurrent illness of mother (No).

Table S7: Output of HTQ-16 model with un-exponentiated estimates.

	Estimate	z.value	95% CI		p-value
			lower	upper	
(Intercept)	0.18	1.13	-0.14	0.51	0.260
Time after discharge from hospital	0.08	2.49	0.02	0.15	0.013
MNM status (near-miss women)	0.13	0.56	-0.32	0.58	0.579
MNM status * time after discharge	-0.10	-2.33	-0.19	-0.02	0.020

Table S8: Main results of negative binomial mixed-effects model for four domains of QoL with un-exponentiated estimates. [a,b,c,d,e]

	Conditional Model					Zero-inflated Model				
			95% CI					95% CI		
	Estimate	z.value	lower	upper	p-value	Estimate	z.value	lower	upper	p-value
**Physical Domain of Quality of Life**										
(Intercept)	4.36	15,67	3,81	4,90	0,000	-8,46	-4,16	-12,45	-4,47	0,000
Time after discharge from hospital	0.00	0,14	-0,03	0,03	0,888	0,01	0,39	-0,06	0,08	0,696
MNM status (near-miss women)	0.19	2,07	0,01	0,37	0,038	-0,48	-1,61	-1,07	0,10	0,107
Intercurrent illness of mother (yes)	0.11	1,43	-0,04	0,26	0,152	-0,90	-3,26	-1,44	-0,36	0,001
Social support scale	-0.07	-9,26	-0,08	-0,05	0,000	0,27	4,33	0,15	0,39	0,000
Mode of delivery (Early pregnancy loss)	0.01	0,14	-0,19	0,22	0,892	0,33	1,00	-0,32	0,98	0,317
Mode of delivery (Cesarean section)	-0.12	-1,55	-0,27	0,03	0,120	0,67	2,72	0,19	1,16	0,006
Age of the women	0.01	2,18	0,00	0,03	0,029	-0,04	-1,86	-0,07	0,00	0,063
MNM status * time after discharge	-0.05	-2,71	-0,08	-0,01	0,007	0,00	-0,06	-0,10	0,10	0,955
**Psychological Domain of Quality of Life**										
(Intercept)	4.53	7,08	3,28	5,79	0,000	-8,08	-2,96	-13,43	-2,73	0,003
Time after discharge from hospital	0.00	-0,03	-0,08	0,08	0,978	-0,07	-0,96	-0,23	0,08	0,339
MNM status (near-miss women)	0.35	1,20	-0,22	0,92	0,228	-0,54	-1,03	-1,57	0,48	0,301
Intercurrent illness of mother (yes)	0.00	-0,02	-0,42	0,42	0,987	-0,48	-1,29	-1,21	0,25	0,196
History of C-Section (Only one)	0.08	0,37	-0,36	0,52	0,711	-1,04	-2,36	-1,91	-0,18	0,018
History of C-Section (two or more)	-0.18	-0,46	-0,96	0,60	0,647	-0,14	-0,21	-1,50	1,21	0,834
Social support scale	-0.07	-3,27	-0,11	-0,03	0,001	0,29	3,31	0,12	0,46	0,001
Residence (Mixed)	-0.45	-1,93	-0,91	0,01	0,053	0,28	0,68	-0,53	1,09	0,493
Residence (Rural)	-0.17	-0,80	-0,60	0,25	0,423	-0,37	-0,86	-1,20	0,47	0,390
Employment (Housewife)	0.05	0,20	-0,44	0,55	0,841	0,51	1,10	-0,40	1,42	0,269
Employment (Self-employed)	0.19	0,66	-0,36	0,74	0,507	0,15	0,28	-0,89	1,19	0,778
Employment (Others)f	1.35	2,50	0,29	2,41	0,013	0,79	0,91	-0,92	2,50	0,365
MNM status * time after discharge	-0.07	-1,34	-0,17	0,03	0,179	0,11	1,17	-0,07	0,29	0,243
**Social Domain of Quality of Life**										
(Intercept)	4.20	28,21	3,91	4,49	0,000	-6,29	-6,39	-8,22	-4,36	0,000
Time after discharge from hospital	-0.01	-0,73	-0,03	0,02	0,464	0,11	3,11	0,04	0,18	0,002

MNM status (near-miss women)	0.02	0,21	-0,13	0,16	0,833	-0,29	-1,08	-0,81	0,23	0,280
Pregnancy outcome (Early pregnancy loss)	-0.13	-1,59	-0,30	0,03	0,113	0,22	0,78	-0,33	0,76	0,438
Pregnancy outcome (Perinatal loss)	0.00	-0,01	-0,14	0,14	0,991	0,48	2,10	0,03	0,93	0,035
Education (Secondary or more)	-0.09	-1,43	-0,20	0,03	0,152	0,33	1,67	-0,06	0,72	0,096
Social support scale	-0.02	-3,92	-0,03	-0,01	0,000	0,19	5,93	0,13	0,25	0,000
MNM status * time after discharge	-0.02	-1,34	-0,06	0,01	0,180	-0,02	-0,40	-0,11	0,07	0,689
Environmental Domain of Quality of Life										
(Intercept)	5.44	33,35	5,12	5,76	0,000	-9,43	-3,42	-14,83	-4,03	0,001
Time after discharge from hospital	-0.03	-3,36	-0,05	-0,01	0,001	0,03	0,70	-0,06	0,13	0,482
MNM status (near-miss women)	0.02	0,34	-0,11	0,16	0,737	-0,08	-0,19	-0,90	0,74	0,851
Intercurrent illness of mother (yes)	0.16	2,89	0,05	0,27	0,004	-0,51	-1,27	-1,30	0,28	0,205
Social support scale	-0.09	-16,54	-0,10	-0,08	0,000	0,21	2,45	0,04	0,38	0,014
Education (Secondary or more)	-0.07	-1,24	-0,17	0,04	0,216	0,86	2,25	0,11	1,61	0,024
MNM status * time after discharge	0.01	0,79	-0,01	0,03	0,432	-0,14	-1,72	-0,30	0,02	0,085

[a] Scores of all domains of QoL are reversely coded in these models, which means zero indicates maximum and 100 the minimum QoL. Therefore, this should be taken into account when interpreting the direction of association of the results in the table.

[b] The output of zero-inflated negative binomial mixed effects analysis has two parts: the negative binomial and the zero inflated part. The zero-inflated part estimates the probability of being exactly zero, like a usual logistic regression model while the negative binomial part estimates the mean score for an individual. (see analysis method section for detail)

[c] The "intercept" shows the initial status (three months) QoL score for a control woman if all other variables are zero. The hypothesis tested here (p-value) is if it is different from zero or not. "Time after discharge from hospital" is the slope (rate of change in QoL) for controls and the hypothesis tested here (p-value) is if the slope is different from zero or not (if there is change overtime or not). While, the interaction between time after discharge and MNM status ("MNM status * time after discharge") indicates if the slope of MNM women differ significantly from the controls. Lastly, "MNM status (near-miss women)" shows if the initial status QoL score of MNM women differ significantly from controls.

[d] The reference category for variables in the model are as follows: MNM status (control women), intercurrent illness of mother (no), mode of delivery (vaginal delivery), history of cesarean section (no previous cesarean section), address (rural), employment (employed), perinatal loss (no), perceived wealth (average), and education (primary or less).

[e] Three months are subtracted from the variable "time after discharge from hospital".

[f] Also include students and non-payed job
